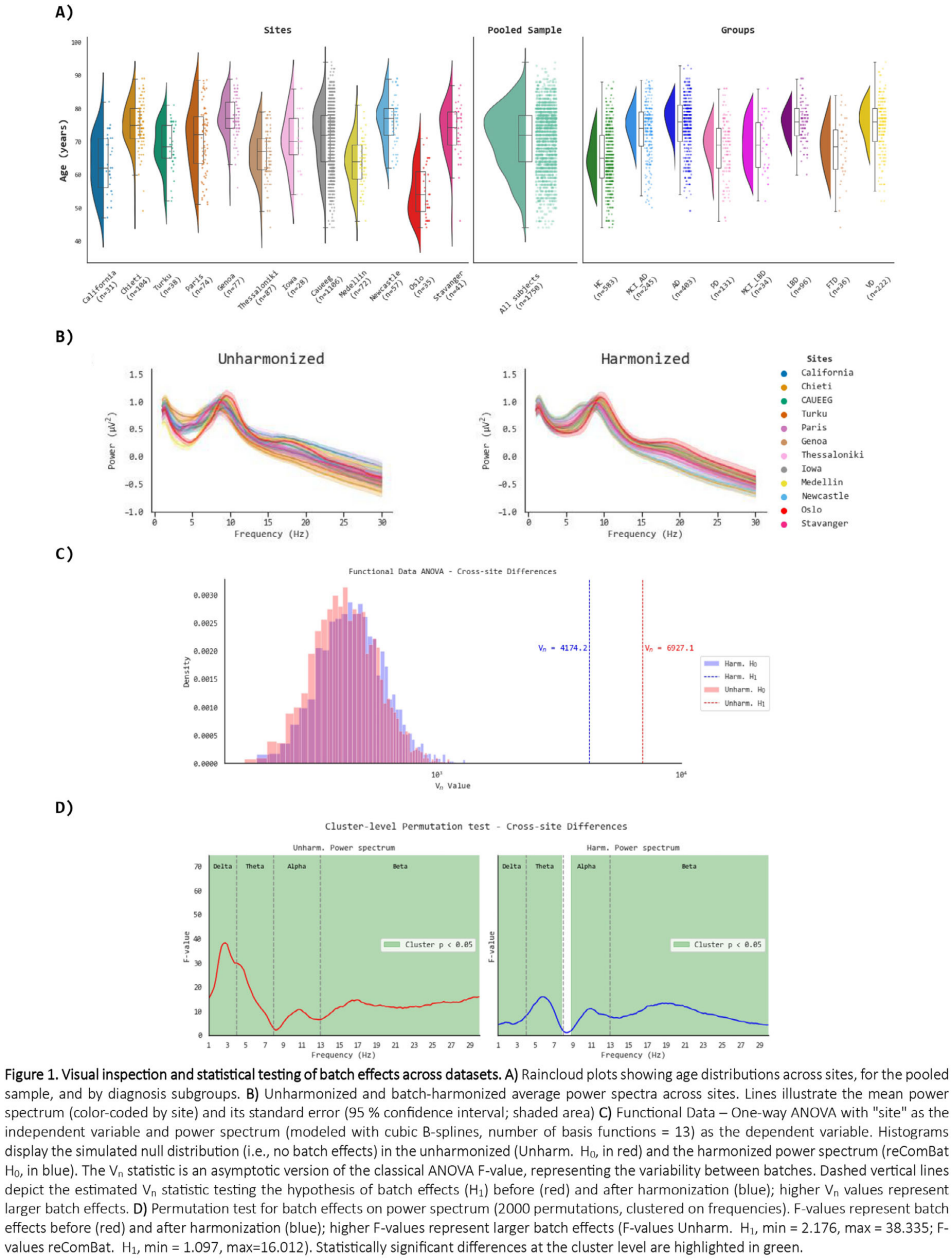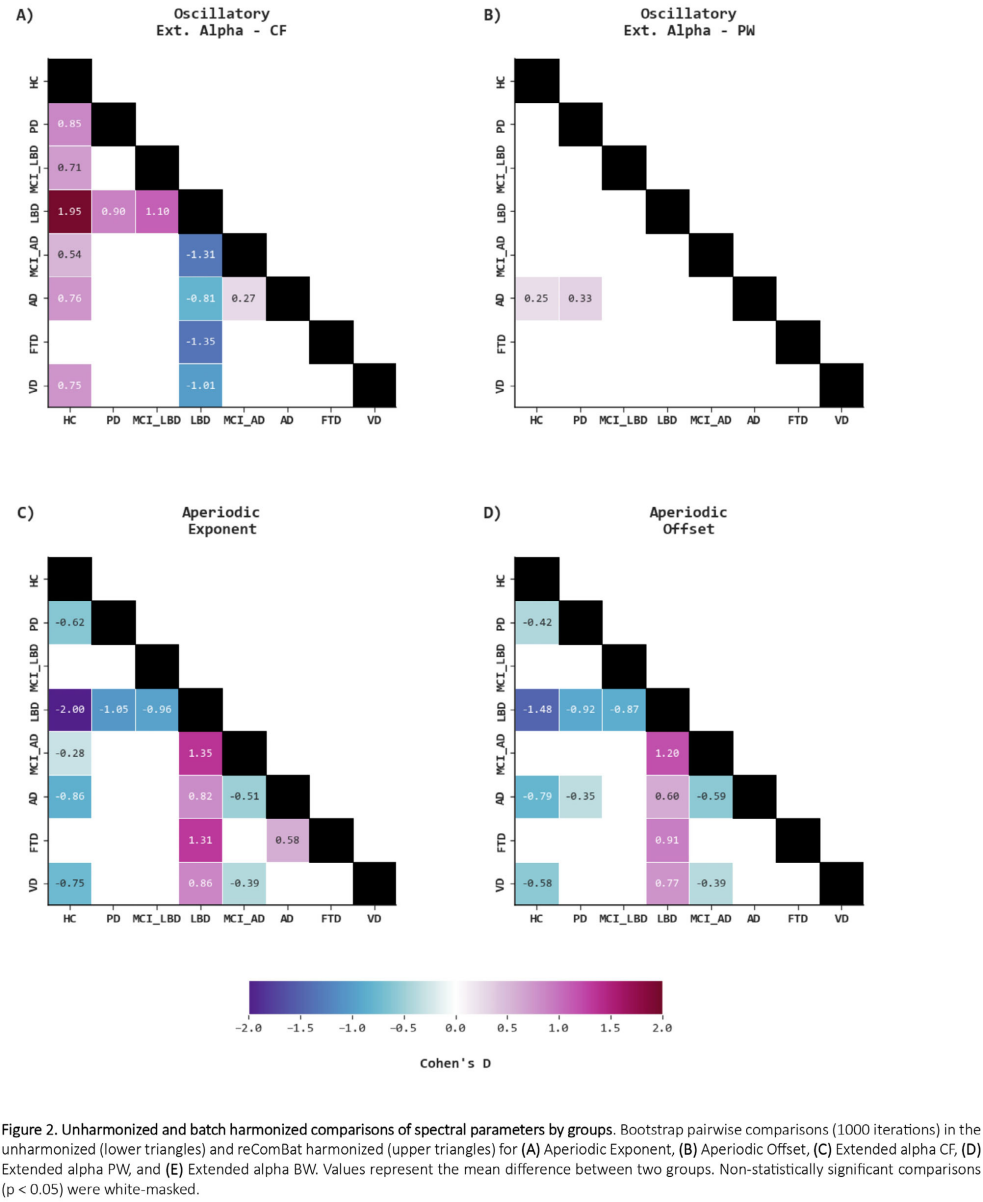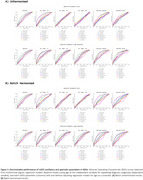# Mega‐Analysis of Oscillatory and Aperiodic Resting‐State EEG Alterations in Neurodegenerative Diseases

**DOI:** 10.1002/alz70856_103714

**Published:** 2025-12-24

**Authors:** Alberto Jaramillo‐Jimenez, Yorguin Jose Mantilla‐Ramos, Diego Tovar, Francisco Lopera, David Fernando Aguillón Niño, John Fredy Ochoa Gómez, Claire Paquet, Sinead Gaubert, Matteo Pardini, Dario Arnaldi, John‐Paul Taylor, Tormod Fladby, Kolbjørn Brønnick, Dag Aarsland, Laura Bonanni, European Dementia with Lewy Bodies Consortium

**Affiliations:** ^1^ Centre for Age‐Related Medicine (SESAM), Stavanger University Hospital, Stavanger, Norway; ^2^ Grupo Neuropsicología y Conducta (GRUNECO), Universidad de Antioquia, Medellín, Colombia; ^3^ Grupo de Neurociencias de Antioquia, Universidad de Antioquia, Medellín, Colombia; ^4^ Semillero de Investigación NeuroCo, Universidad de Antioquia, Medellín, Colombia; ^5^ Cognitive and Computational Neuroscience Laboratory (CoCo Lab), University of Montreal, Montreal, Canada; ^6^ Doctoral School Biomedical Sciences, KU Leuven, Leuven, Belgium; ^7^ Grupo de Neurociencias de Antioquia, Universidad de Antioquia, Medellín, Antioquia, Colombia; ^8^ Grupo Neuropsicología y Conducta (GRUNECO), Universidad de Antioquia, Medellín, Antioquia, Colombia; ^9^ Semillero Neurociencias Computacionales NeuroCo, Medellín, Antioquia, Colombia; ^10^ Therapeutic Optimization in Neuropsychopharmacology, Université Paris Cité, Paris, France; ^11^ Cognitive Neurology Center, GHU.Nord APHP Hôpital Lariboisière FW, Paris, France; ^12^ IRCCS Ospedale Policlinico San Martino, Genova, Italy; ^13^ DINOGMI, Università degli studi di Genova, Genova, Italy; ^14^ Translational and Clinical Research Institute, Newcastle University, Newcastle upon Tyne, United Kingdom; ^15^ Akershus University Hospital, Nordbyhagen, Norway, Norway; ^16^ Institute of Clinical Medicine, University of Oslo, Oslo, Norway; ^17^ Faculty of Social Sciences, University of Stavanger, Stavanger, Norway; ^18^ Institute of Psychiatry, Psychology & Neuroscience, King's College London, London, United Kingdom; ^19^ Department of Medicine and Aging Sciences, University G. d’Annunzio of Chieti‐Pescara, Chieti, Chieti, Italy

## Abstract

**Background:**

Resting‐state EEG (rsEEG) alterations in the posterior alpha rhythm are promising biomarkers of neurodegenerative diseases (NDDs). However, spectral analysis often overlooks the rsEEG non‐rhythmic (aperiodic) component. Studies assessing the oscillatory and aperiodic activity are scarce and frequently underpowered. While multicenter data pooling (mega‐analysis) can enhance statistical power, it may introduce site‐related differences (batch effects). This mega‐analysis differentiates rsEEG oscillatory and aperiodic alterations across NDDs while mitigating batch effects.

**Method:**

RsEEGs from 1750 subjects across 12 sites were preprocessed. We pooled signals from healthy controls (HC = 583), Parkinson's Disease (PD = 131), Lewy Body Dementias (LBD = 96), Alzheimer's Disease (AD = 403), Frontotemporal Dementia (FTD = 36), Mild Cognitive Impairment (MCI) in Lewy Bodies pathology or PD (MCI‐LBD = 34), MCI in AD spectrum (MCI‐AD = 245), and Vascular Dementia (VD = 222); Figure 1A. Batch effects harmonization of the posterior power spectrum was performed with reComBat (age and diagnosis‐adjusted). We evaluated harmonization through functional and mass‐univariate permutation ANOVAs. Oscillatory and aperiodic parameters were extracted from the harmonized spectrum with specparam. Group differences across NDDs were estimated with bootstrap pairwise comparisons, mass‐univariate permutation tests, and logistic regression models (age‐adjusted).

**Result:**

Visualizations and statistical testing supported reduced batch effects after harmonization; Figure 1B and 1C. As consistent results in the unharmonized and harmonized data, steeper aperiodic parameters and lower oscillatory center frequency characterized LBD compared to all other groups. Besides, oscillatory extended alpha power was lower in AD than in HC and PD; Figure 2. Harmonized oscillatory center frequency and aperiodic parameters improved the separation of LBD compared to unharmonized parameters; Figure 3.

**Conclusion:**

Harmonization mitigates batch effects in the rsEEG posterior power spectrum. LBD is characterized by pronounced oscillatory frequency slowing and increased aperiodic activity, while AD displays both oscillatory and aperiodic abnormalities with smaller effect sizes.